# UPLC-MS/MS Method for Simultaneous Determination of 14 Antimicrobials in Human Plasma and Cerebrospinal Fluid: Application to Therapeutic Drug Monitoring

**DOI:** 10.1155/2022/7048605

**Published:** 2022-01-05

**Authors:** Huiting Sun, Han Xing, Xueke Tian, Xiaojian Zhang, Jing Yang, Peile Wang

**Affiliations:** ^1^Department of Pharmacy, First Affiliated Hospital of Zhengzhou University, Zhengzhou 450052, China; ^2^Henan Key Laboratory of Precision Clinical Pharmacy, Zhengzhou 450052, China

## Abstract

Pharmacokinetics/pharmacodynamics is the foundation for guiding the rational application of antibiotics in clinical practice, so it is necessary to establish quantitative methods for accurate drug concentration determination. This study aimed to develop a rapid and simple ultrahigh-performance liquid chromatography-tandem mass spectrometry (UPLC-MS/MS) method for simultaneous quantification of 14 antibiotics (amikacin, etimicin, ceftazidime, cefepime, cefoperazone, ceftriaxone, daptomycin, latamoxef, linezolid, meropenem, biapenem, ampicillin, norvancomycin, and vancomycin) in human plasma and cerebrospinal fluid. Antibiotics were chromatographically separated on a Waters ACQUITY UPLC BEH C18 column (2.1 mm × 50 mm, 1.7 *μ*m) via gradient elution within 3 minutes and were monitored using positive ion fitted with multiple reaction monitoring. The lower limit of quantification was 0.05–2.0 *μ*g·mL^−1^. The method was verified according to the FDA bioanalysis method validation guidelines, which showed excellent accuracy (from 86.75% to 110.85%) and precision (from 0.46% to 10.97%). At last, this method was successfully applied to therapeutic drug monitoring in 113 patients under antibiotics treatment.

## 1. Introduction

Central nervous system (CNS) infection is one of the most serious infections. The blood-brain barrier surrounding CNS and the emergence of multiple drug-resistant bacteria in recent years pose a therapeutic challenge for treating CNS infections [1, 2]. As pharmacokinetics/pharmacodynamics (PK/PD) is characteristic of antimicrobial agents, the Infectious Diseases Society of America's Clinical Practice Guidelines recommended that dosages of intracranial antimicrobial therapy should be adjusted based on the cerebrospinal fluid (CSF) concentrations of antibiotics to 10–20 times the minimum inhibitory concentration of the causative microorganism [3]. Accordingly, individualizing antibiotic dosing via therapeutic drug monitoring (TDM) should be considered to maximize therapeutic success and reduce the generation of resistant bacteria [4, 5]. Therefore, it is necessary to establish a reliable quantitative method to monitor the antibiotic concentrations in plasma and CSF.

In recent years, several assays mainly using liquid chromatography combined with ultraviolet (LC-UV) or mass spectrometry (LC-MS) detection have been developed [6–9]. Most methods were based on time-consuming sample preparation procedures, covered a few or a class of antibiotics, and only measured drug concentration in blood or long analysis time [10–15]. In the clinic, due to the high mortality of CNS infection and low positive rate of bacterial culture, combination therapy is widely used to prevent or control CNS infection, and drug concentration at the infection site was directly related to clinical efficacy and adverse reactions [3]. These methods, which only measure one or a class of antibiotics in the blood, are insufficient to meet clinical needs. Therefore, it is necessary to develop a simple and rapid method to cover frequently used antibiotics in both blood and infection sites to assist TDM in routine laboratory practice.

The aim of this study was to develop and validate an LC-MS method for simultaneous determination of 14 antibiotics frequently used for the treatment of CNS bacterial infections (amikacin, etimicin, ceftazidime, cefepime, cefoperazone, ceftriaxone, daptomycin, latamoxef, linezolid, meropenem, biapenem, ampicillin, norvancomycin, and vancomycin) in human plasma and CSF. After meeting all the requirements in the bioanalytical guidance, the approach was applied for the TDM of antibiotics in patients, especially those with central system infection.

## 2. Materials and Methods

### 2.1. Reagents and Chemicals

Meropenem (Lot: 130508-201403), norvancomycin (Lot: 130338-201704), ampicillin (Lot: 130410-201908), linezolid (Lot: AL190827-16), ceftazidime (Lot: 130484-201806), ceftriaxone (Lot: 130480-201504), cefoperazone (Lot: 130420-201105), vancomycin (Lot: 130360-201302), cefepime (Lot: 130524-201404), biapenem (Lot: B-03000-04), latamoxef (Lot: 130590-201702), and etimicin (Lot: 130551-201902) were purchased from China Institute for Food and Drug Control. Daptomycin (Lot: LAC0T96) was purchased from Beijing Bailingwei Technology Co., Ltd. (Beijing, China). Amikacin (Lot: 130335-200204), linezolid-*d*3 (Lot: 25-NOV-18-67), meropenem-*d*6 (Lot: M225617), and ceftriaxone-d3 (Lot: 19-MRA-19-46) were purchased from China Institute for the Control of Pharmaceutical and Biological Products. The last three were internal standards (IS).

LC-MS grade methanol and acetonitrile were bought from Thermo Fisher Technology Co., Ltd. (Shanghai, China), and distilled water was purchased from Guangzhou Watsons Food and Beverage Co., Ltd. (Guangzhou, China).

### 2.2. LC-MS/MS Analysis

Chromatographic analysis was performed on an ACQUITY I-Class UPLC and a XEVO TQD triple quadrupole mass spectrometer equipped with an electrospray ionization interface (Waters, Milford, MA). Chromatographic separation was performed on a Waters Van Guard BEH C_18_ column (2.1 mm × 50 mm, 1.7 *μ*m). The column temperature was maintained at 40°C. The autosampler temperature was set at 8°C. The mobile phase, consisting of eluents *A* (0.1% formic acid (FA) in water, *v/v*) and *B* (0.1% FA in methanol, *v/v*), was delivered at a flow of 0.4 mL/min using a linear gradient program as follows: 2%–35% from 0 to 0.4 minutes and 35%–98% from 0.4 to 2.0 minutes. Posttime was set at 1.0 min for the mobile phase to revert to the initial 2% *B* before the next injection.

Mass analysis was designed as follows: capillary voltage, 3.0 kV; source temperature, 150°C; desolvation temperature, 500°C; cone gas flow, 50 L/h; and desolvation gas flow, 800 L/h; solvent removal and cone hole reverse blowing gas was nitrogen; collision gas was argon; scanning mode was multireaction monitoring; the main parameters are shown in Table 1. Data processing was performed using MassLynx 4.0 Software (Waters, Milford, MA).

### 2.3. Preparation of Standards, Calibrators, and Quality Control (QC) Samples

To prepare the stock solutions, about 2 mg (biapenem and ceftriaxone) or 10 mg (amikacin, etimicin, ceftazidime, cefepime, cefoperazone, daptomycin, latamoxef, linezolid, meropenem, ampicillin, norvancomycin, and vancomycin) were dissolved in 5% methanol-water (*v/v*), except for ceftriaxone and daptomycin that were dissolved in 50% methanol-water (*v/v*).

The first calibration curve (containing ceftazidime, ceftriaxone, daptomycin, linezolid, meropenem, ampicillin, and norvancomycin) and the second calibration curve (containing latamoxef, vancomycin, biapenem, amikacin, etimicin, cefepime, and cefoperazone) were prepared fresh daily by dissolving the stock solutions in both blank plasma and CSF, respectively. The calibration standards, the limit of quantitation (LLOQ), and low, medium, and high quality control (LQC, MQC, and HQC) are shown in Tables 2 and 3. The stock solution of three IS (1.0 mg mL^−1^, in 50% methanol-water, *v/v*) was diluted with acetonitrile-methanol (3 : 1, *v/v*) to obtain the precipitating agent with a final IS concentration of 7.5 *μ*g·mL^−1^. All solutions were stored at −80°C and were brought to room temperature before use.

### 2.4. Sample Preparation

100 *μ*L plasma or CSF sample was mixed with a 300 *μ*L precipitating agent. After a thorough vortex for 1 min, the mixture was centrifuged at 13,300 × *g* for 15 min at 4°C. 100 *μ*L supernatant was then transferred to a 96-well plate, and 2.5 *μ*L supernatant was injected into LC-MS for analysis.

### 2.5. Method Validation

The method validation, including selectivity, linearity, accuracy, precision, matrix effects, extraction recovery, stability, and carryover, was performed in accordance with the latest USA Food and Drug Administration bioanalytical method validation guidance [16].

Selectivity was performed with six blank plasma or CSF samples from different individual donors and chromatograms of blank plasma or CSF were compared with the corresponding spiked plasma or CSF samples. The interference peak area of analytes in blank samples should be less than 20% of LLOQ and <5% of IS.

Calibration curves were freshly prepared and evaluated during three different working days. The peak area ratios of each analyte/IS (*y*) versus nominal concentrations (*x*) were fitted into a linear regression curve with 1/*x*-weighted or 1/*x*^2^-weighted. 75% calibrator levels should be within 85%–115% of their theoretical concentration, while they were within 80%–120% for LLOQ. As for carryover, a blank sample was set after three upper limits of quantitation (ULOQ) to investigate the systematic residue of the analytes.

Accuracy (% bias) and precision (RSD) were evaluated using QC samples at three concentration levels (LQC, MQC, and HQC). The intraday precision and accuracy were assessed by six replicate samples for each concentration level analyzed within one day, and the interday precision and accuracy were assessed within three successive days. The acceptance value for precision and accuracy should fall within ±15%. For LLOQ, the precision and accuracy should be less than ±20%.

The matrix effect and recovery were evaluated at three sets of QC samples. Set *A*: QCs were extracted as Section 2.4 to obtain peak area *A*1. Set *B*: analytes were diluted with 5% methanol-water (*v/v*) instead of blank plasma or CSF and then extracted as shown in Section 2.4 to obtain peak area *A*2. Set *C*: analytes were added to the extracted blank matrix and then extracted as shown in Section 2.4 to obtain peak area *A*3. Matrix effect was calculated as *A*3/*A*2 × 100%, and recovery was calculated as *A*1/*A*3 × 100%.

The stability of analytes was evaluated in triplicate at LQC and HQC. Samples were stored at room temperature (24°C) for 24 hours, frozen at −20°C for 14 days, frozen-thawed three times, and frozen at −80°C for 30 days to investigate short-term stability and long-term stability, respectively.

### 2.6. Application to Clinical Samples

Plasma and CSF concentrations from routine TDM at the First Affiliated Hospital of Zhengzhou University from January 2021 and June 2021 were retrospectively collected and reanalyzed by this new LC-MS/MS method. Demographic characteristics and anti-infection treatment were collected from electronic medical records and anonymized. The study protocol was approved by the hospital ethics committee review board (Zhengzhou University Medical Research and Ethics Committee, No. 2021-KY-0425), and written informed consent was not required. Blood and CSF samples were centrifuged at 3,500 × *g* for 10 min at 4°C, and the supernatant was collected and stored at −80°C until analysis.

## 3. Results and Discussion

### 3.1. Method Development

According to the clinical practice, 14 commonly used antibiotics in CNS bacterial infection, including eight *β*-lactams (ampicillin, ceftazidime, ceftriaxone, cefepime, cefoperazone, meropenem, biapenem, and latamoxef), two aminoglycosides (amikacin and etimicin), three glycopeptides (vancomycin, norvancomycin, and linezolid), and one cyclic lipopeptide (daptomycin), were selected for simultaneous determination in plasma and CSF samples. Due to the different types and water solubility of antibiotics, it was challenging to develop a simple quantification method for all analytes.

For mass conditions, electrospray ionization (ESI) in positive mode was selected for scanning all analytes. Table 1 displays relevant LC-MS/MS characteristics and Figure S1 depicts mass spectrums of 14 antibiotics. To optimize chromatographic conditions, mobile phase (water-methanol vs. water-acetonitrile), buffer compositions (ammonium formate vs. formic acid), buffer concentration (0.1–0.2%, *v*/*v*), flow rate (0.2–0.4 mL/min), and injection volume (2.5–10 *μ*L) under a variety of gradients were tested for method robustness. As a result, final chromatographic conditions were set as follows: 0.1% FA in water−0.1% FA in methanol was delivered at a flow of 0.4 mL/min using a linear gradient program with a 2.5 *μ*L injection volume.

Regarding the biological sample preprocessing methods, a simple protein precipitation method with different organic solvents was tested. The results showed that the protein was precipitated completely with acetonitrile, while the high mass spectrometric response of etimicin and amikacin was observed with methanol. Therefore, the mixture of acetonitrile-methanol (3 : 1, *v/v*) was chosen to precipitate protein.

### 3.2. Method Validation

#### 3.2.1. Selectivity, Linearity, and Carryover

As shown in Figures S1 and S2, analyte peaks were detected with excellent resolution and shapes. In the blank samples, there was no observed significant interference at the analyte retention times, which indicated that this method had sufficient selectivity. Based on the published literature on clinical study, the linear range of 14 antibiotics was shown in Table 2 [4, 17, 18]. Linezolid-*d*3 was used as the IS of linezolid, ampicillin, ceftazidime, daptomycin, latamoxef, biapenem, and cefoperazone; meropenem-*d*6 as the IS of meropenem, vancomycin, norvancomycin, cefepime, amikacin, and etimicin; and ceftriaxone-*d*3 as the IS of ceftriaxone. The linearity of 14 antibiotics was assessed over the plasma therapeutic range with a regression coefficient *r* ≥ 0.99. No remarkable carryover residue in blank samples following three ULOQ samples was found under the LC-MS/MS conditions.

#### 3.2.2. Accuracy, Precision, and LLOQ

As shown in Table 3, the intra- and interassay accuracies of 14 antimicrobials ranged from 86.75% to 110.85%, and the RSD of intra- and interbatch precision was all less than 10.97%. The accuracy of LLOQ in plasma and CSF was ranged from 83.93% to 118.88%, and the RSD of precision was less than 13.72%. The results indicated that this method was accurate and reliable (Table S1).

#### 3.2.3. Matrix Effects and Recovery

Table 4 presents the recovery and matrix effects of analytes. The RSD of matrix effects derived from QC samples was below 14.41%. The RSD of recovery was between 1.20% and 14.63%, which met the criteria.

#### 3.2.4. Stability

As shown in Tables S2 and S3, the accuracy of LQC and HQC at different conditions was 86.40%–113.82% with RSD less than 13.93%, which indicated 14 analytes were stable in human plasma and CSF at room temperature for 24 hours, which were frozen at 20°C for 14 days, frozen-thawed three times, and frozen at −80°C for 30 days.

### 3.3. Application to Clinical Samples

In total, this LC-MS/MS method was applied to 125 blood samples and 10 CSF samples collected from 9 pediatric and 104 adult patients. All TDM samples were collected before the next dose at a steady state (*C*_min_). Data concerning TDM of seven antibiotics are summarized in Table 5, which were similar to the data in reports from the nosocomial clinical laboratory and previous literature [4, 17, 18]. *C*_min_ of meropenem, linezolid, and vancomycin varied greatly among individuals, and two plasma trough concentrations of meropenem were below LLOQ. Due to the large individual variability, further research should be performed on the PK/PD and dose optimization of antibiotics.

At present, several LC-MS methods have been developed to simultaneously quantify antibacterial agents [6–15]. This method presented multiple advantages compared to the above methods. First, the use of UPLC-MS/MS allows for better selectivity and a shorter analysis time than HPLC and higher sensitivity and less interference from other endogenous substances or metabolites than UV detection [6, 7]. Second, the determination of 14 antibiotics covering almost all antibiotics used to treat CNS bacterial infections was suitable for routine clinical use. Simultaneous determination of 14 antibiotics in both CSF and plasma could provide the actual concentrations of infection sites, which will assist the clinician in making an optimal decision.

## 4. Conclusions

This simple and rapid LC-MS/MS method was developed and validated for simultaneous measurement of 14 antibiotics in CSF and plasma. It was suitable for the TDM of antibiotic therapy in critically ill patients, particularly those with CNS bacterial infection.

## Figures and Tables

**Table 1:** Acquisition parameters used in the UPLC-MS/MS assay.

Compounds	Parent (*m*/*z*)	Daughter (*m*/*z*)	RT (min)	Dwell (s)	Cone (V)	Collision (V)
Meropenem	384.1	141.1	0.81	0.015	27	14
Norvancomycin	718.0	144.1	0.74	0.039	30	20
Ampicillin	350.1	106.1	1.01	0.039	28	16
Linezolid	338.1	296.0	1.18	0.039	46	22
Ceftazidime	547.0	468.0	0.81	0.039	30	12
Ceftriaxone	555.0	395.9	0.93	0.039	30	12
Daptomycin	810.7	159.1	1.62	0.120	38	56
Vancomycin	725.6	144.1	0.76	0.038	35	16
Cefepime	481.0	125.0	0.73	0.038	28	52
Biapenem	351.1	110.1	0.70	0.038	34	18
Cefoperazone	647.0	530.9	1.02	0.038	35	15
Latamoxef	521.0	377.0	0.91	0.038	24	24
Etimicin	478.1	350.1	0.27	0.052	42	24
Amikacin	586.2	163.1	0.27	0.052	40	36
Linezolid-*d*3	341.1	296.0	1.18	0.039	46	22
Ceftriaxone-*d*3	558.0	395.9	0.93	0.039	30	12
Meropenem-*d*6	390.2	146.9	0.81	0.015	27	14

RT: retention time.

**Table 2:** Concentrations of calibrators (*μ*g/mL) and regression equation.

Compounds	Calibration curve 1								Regression equation
Meropenem	0.5	1	2.5	5	10	25	50	100	*Y* = 3.352*X* − 0.0918
Norvancomycin	0.5	1	2.5	5	10	25	50	100	*Y* = 0.0565*X* − 0.00848
Ampicillin	0.5	1	2.5	5	10	25	50	100	*Y* = 5.671*X* − 2.0614
Linezolid	0.25	0.5	1.25	2.5	5	12.5	25	50	*Y* = 2.0248*X* + 0.000798
Ceftazidime	0.4	0.8	2	4	8	20	40	80	*Y* = 0.285*X* − 0.0685
Ceftriaxone	0.05	0.1	0.25	0.5	1	2.5	5	10	*Y* = 2.589*X* − 0.0144
Daptomycin	0.5	1	2.5	5	10	25	50	100	*Y* = 2.174*X* − 0.352

	Calibration curve 2								

Vancomycin	0.5	0.8	2.5	5	12.5	25	50	—	*Y* = 0.0265*X* + 0.00375
Cefepime	0.5	0.8	2.5	5	12.5	25	50	—	*Y* = 0.0885*X* − 0.0232
Biapenem	0.25	0.4	1.25	2.5	6.25	12.5	25	—	*Y* = 0.0189*X* − 0.000895
Cefoperazone	0.2	0.3	1	2	5	10	20	—	*Y* = 0.0811*X* − 0.00720
Latamoxef	0.6	1	3	6	15	30	60	—	*Y* = 0.104*X* − 0.00349
Etimicin	—	0.65	2	4	10	20	40	—	*Y* = 0.0318*X* − 0.00469
Amikacin	—	1.3	4	8	20	40	80	—	*Y* = 0.00335*X* − 0.00102

**Table 3:** Precision and accuracy for 14 antibiotics in human plasma and cerebrospinal fluid (*n* = 6).

Compounds	Spiked (*μ*g/mL)	Plasma			Cerebrospinal fluid		
		Interday precision RSD (%)	Intraday accuracy (%)	Intraday precision RSD (%)	Interday precision RSD (%)	Intraday accuracy (%)	Intraday precision RSD (%)
Meropenem	1.5	2.52	109.49	2.82	2.32	99.84	3.08
	20	2.51	108.99	2.86	3.03	103.13	4.83
	80	3.34	105.49	4.59	3.91	103.37	4.15

Norvancomycin	1.5	4.72	101.77	4.14	2.88	103.00	1.67
	20	5.78	108.53	6.14	2.30	99.25	2.50
	80	4.77	105.73	3.13	1.72	108.81	2.20

Ampicillin	1.5	1.60	104.32	1.96	3.81	103.03	1.28
	20	2.36	101.77	2.36	2.39	99.69	1.50
	80	2.77	104.97	3.72	1.64	107.95	1.80

Linezolid	0.75	1.81	105.40	2.01	1.47	101.00	1.08
	10	1.63	107.17	2.35	1.41	105.77	1.05
	40	2.88	103.76	3.20	1.71	102.39	0.95

Ceftazidime	1.2	2.50	93.83	1.94	2.57	96.49	3.58
	16	2.31	98.73	2.18	3.34	101.02	4.23
	64	3.11	104.42	2.99	2.23	106.93	2.63

Ceftriaxone	0.15	2.89	108.33	3.43	5.17	99.67	5.90
	2	3.72	99.82	5.05	3.81	106.81	4.56
	8	3.84	92.15	2.98	4.79	96.58	2.44

Daptomycin	1.5	1.58	99.42	1.64	3.20	95.58	1.68
	20	1.52	110.85	2.35	1.63	107.00	1.68
	80	2.04	107.29	1.16	1.65	109.15	1.44

Vancomycin	1	8.71	101.07	10.97	6.42	103.35	7.37
	10	6.05	100.86	8.38	5.55	104.99	7.80
	40	4.97	96.11	10.53	3.69	106.54	4.46

Cefepime	1	6.14	100.57	8.22	2.87	110.85	2.64
	10	3.32	102.47	5.85	5.39	98.04	5.67
	40	3.15	103.03	1.38	2.80	110.15	3.35

Biapenem	0.5	3.17	105.30	2.56	5.07	101.57	7.07
	5	3.64	107.97	5.77	3.07	101.14	3.44
	20	3.95	101.89	5.34	2.44	96.93	2.19

Cefoperazone	0.4	3.43	97.79	2.62	2.58	107.08	2.37
	4	1.72	106.71	1.83	1.90	91.27	1.97
	16	2.83	102.19	4.26	2.63	104.84	5.26

Latamoxef	1.2	2.87	97.94	3.53	2.56	101.58	2.17
	12	1.32	109.92	1.27	2.00	104.31	2.75
	48	3.15	107.96	4.30	1.51	103.57	2.08

Etimicin	1.6	4.75	86.75	1.97	4.66	100.90	8.40
	8	2.82	87.85	3.67	3.46	104.67	2.45
	32	3.05	107.15	3.62	4.36	100.40	7.41

Amikacin	3.2	5.27	93.15	4.08	0.47	91.52	0.46
	16	6.10	103.80	7.80	2.27	89.36	1.51
	64	5.75	97.86	3.79	5.77	104.77	3.19

**Table 4:** Matrix effects and recoveries of 14 antibiotics in plasma and cerebrospinal fluid (*n* = 6).

Compounds	Spiked (*μ*g/mL)	Plasma				Cerebrospinal fluid			
		Matrix effect		Recovery		Matrix effect		Recovery	
		Accuracy (%)	RSD (%)	Accuracy (%)	RSD (%)	Accuracy (%)	RSD (%)	Accuracy (%)	RSD (%)
Meropenem	1.5	81.27	11.61	64.29	7.67	64.48	11.62	64.37	12.51
	20	83.82	5.12	64.91	6.90	77.72	8.96	77.65	8.08
	80	82.03	3.39	71.97	2.51	68.24	3.58	70.21	4.65

Norvancomycin	1.5	110.29	11.66	26.40	12.19	88.92	6.56	52.07	14.20
	20	114.07	7.48	29.46	11.28	97.70	3.56	65.48	4.55
	80	114.72	1.44	31.90	6.29	89.96	5.65	67.79	5.28

Ampicillin	1.5	58.04	13.09	59.41	4.0	72.89	11.41	74.87	10.06
	20	77.29	3.17	63.72	5.11	95.01	6.30	75.11	9.65
	80	87.83	3.41	77.70	1.56	88.59	8.40	87.99	8.74

Linezolid	0.75	64.01	5.85	72.29	4.90	63.49	11.54	53.34	13.51
	10	79.68	7.23	74.53	8.43	86.76	2.04	69.91	2.32
	40	82.14	7.78	78.90	4.84	89.21	2.79	74.05	2.64

Ceftazidime	1.2	44.14	14.22	74.96	10.89	75.68	8.86	76.01	5.05
	16	45.46	2.26	69.68	6.42	93.28	14.41	84.78	13.66
	64	51.83	2.51	70.27	3.75	60.33	4.50	53.02	4.28

Ceftriaxone	0.15	53.12	11.96	49.90	12.98	62.13	14.33	66.74	14.63
	2	46.33	4.86	64.37	8.06	62.64	6.29	67.45	7.62
	8	49.55	4.11	68.12	4.56	52.86	5.38	62.25	5.94

Daptomycin	1.5	38.08	10.47	113.94	6.41	40.84	13.30	105.45	12.38
	20	43.15	4.31	95.49	2.87	65.50	7.58	106.07	8.63
	80	51.61	6.29	85.35	1.20	62.62	3.34	79.53	2.57

Vancomycin	1	81.60	10.70	70.78	6.51	51.05	10.44	39.33	12.43
	10	82.11	6.38	69.32	6.73	73.99	8.16	43.99	12.99
	40	83.84	10.38	60.81	11.87	84.62	5.44	51.32	5.96

Cefepime	1	98.78	12.58	91.97	9.23	89.68	10.32	85.12	6.75
	10	79.68	13.42	98.09	7.82	64.86	8.41	62.67	3.97
	40	90.35	4.08	86.59	6.05	73.11	2.44	113.35	3.52

Biapenem	0.5	78.30	5.53	67.67	14.46	42.65	8.01	82.90	5.96
	5	77.64	13.31	94.25	5.96	48.78	3.54	80.04	4.65
	20	67.15	4.21	76.04	9.17	60.81	1.76	89.91	2.25

Cefoperazone	0.4	69.40	6.80	77.94	12.76	83.74	6.58	107.18	4.16
	4	86.58	2.65	75.69	2.70	85.10	1.42	113.51	7.00
	16	94.99	5.31	67.85	5.05	83.88	2.33	95.48	6.39

Latamoxef	1.2	80.98	8.43	71.35	9.40	87.96	13.65	51.56	11.00
	12	97.79	3.14	64.59	4.28	108.96	13.99	73.15	14.59
	48	99.55	1.58	66.27	3.45	103.79	10.47	62.15	8.75

Etimicin	1.6	98.00	8.78	75.25	6.85	98.89	6.01	100.38	9.18
	8	94.96	3.52	94.80	8.51	113.02	3.10	97.25	3.16
	32	94.32	4.44	88.27	5.37	83.48	2.65	114.49	2.76

Amikacin	3.2	99.91	3.68	107.03	13.64	90.74	10.96	98.87	2.53
	16	87.05	7.32	114.77	6.48	70.94	8.04	100.45	3.99
	64	99.56	7.76	110.16	10.32	99.41	2.29	99.31	2.37

**Table 5:** Results of the measurement of antibiotics from patients' samples.

Analytes	Plasma samples (*n*)	CSF samples (*n*)	Patients (*n*)	Mean concentration (range, *μ*g/mL)	Number below LLOQ (%)
Meropenem	23	3	17	13.14 (0.14–74.70)	2 (7.6)
Ampicillin	1	0	1	2.75	0 (0)
Linezolid	17	1	17	11.82 (0.59–42.38)	0 (0)
Ceftazidime	1	1	2	8.52 (1.4–15.64)	0 (0)
Vancomycin	73	4	65	14.38 (0.91–37.97)	0 (0)
Cefepime	4	1	5	27.92 (2.12–49.35)	0 (0)
Biapenem	6	0	6	1.72 (0.25–4.91)	0 (0)
Total	125	10	113		2 (1.4)

## Data Availability

The data used to support the findings of this study are available from the corresponding author upon request.

## Supplementary Materials

Supplementary Materials Table S1: LLOQ of 14 antibiotics in human plasma and cerebrospinal fluid (*n* = 6). Table S2: stability of 14 antibiotics in human plasma (*n* = 3). Table S3: stability of 14 antibiotics in human cerebrospinal fluid (*n* = 3). Figure S1: acquisition parameters used in the UPLC-MS/MS assay. Figure S2: total ion chromatogram of blank sample and analytes.
