# Supplementary material for: UPLC-MS/MS Method for Simultaneous Determination of 14 Antimicrobials in Human Plasma and Cerebrospinal Fluid: Application to Therapeutic Drug Monitoring
Source: J Anal Methods Chem. 2022 Jan 5;2022:7048605. doi: 10.1155/2022/7048605 (PMC8754666; doi:10.1155/2022/7048605)
Supplement: Supplementary Materials — Table S1: LLOQ of 14 antibiotics in human plasma and cerebrospinal fluid (n = 6). Table S2: stability of 14 antibiotics in human plasma (n = 3). Table S3: stability of 14 antibiotics in human cerebrospinal fluid (n = 3). Figure S1: acquisition parameters used in the UPLC-MS/MS assay. Figure S2: total ion chromatogram of blank sample and analytes. [file 7048605.f1.doc]

**Table S1** LLOQ of 14 antibiotics in human plasma and cerebrospinal fluid (n=6).

| **Compounds** | **Spiked**  **(μg/mL)** | **plasma** | | **cerebrospinal fluid** | |
| --- | --- | --- | --- | --- | --- |
|  |  | **Accuracy (%)** | **RSD (%)** | **Accuracy (%)** | **RSD (%)** |
| Meropenem | 0.5 | 103.17 | 2.47 | 97.73 | 5.48 |
| Norvancomycin | 0.5 | 104.30 | 3.27 | 108.00 | 5.74 |
| Ampicillin | 0.5 | 117.13 | 1.51 | 117.57 | 0.89 |
| Linezolid | 0.25 | 97.87 | 1.20 | 100.33 | 1.88 |
| Ceftazidime | 0.4 | 111.25 | 2.36 | 118.88 | 0.95 |
| Cefriaxone | 0.05 | 101.00 | 9.43 | 101.67 | 9.91 |
| Daptomycin | 0.5 | 109.73 | 1.39 | 107.20 | 0.99 |
| Vancomycin | 0.5 | 90.90 | 7.48 | 83.93 | 4.90 |
| Cefepime | 0.5 | 89.03 | 4.08 | 89.33 | 4.07 |
| Biapenem | 0.25 | 99.53 | 12.13 | 99.53 | 12.12 |
| Cefoperazone | 0.2 | 117.17 | 1.54 | 117.17 | 1.54 |
| Latamoxef | 0.6 | 110.36 | 4.40 | 106.56 | 2.31 |
| Etimicin | 0.65 | 95.28 | 13.72 | 104.08 | 8.41 |
| Amikacin | 1.3 | 117.38 | 0.84 | 117.47 | 0.75 |

LLOQ: lower limits of quantifications.

**Table S2** Stability of 14 antibiotics in human plasma (n=3).

| **Compounds** | | **room temperature for 24 h** | | **-20 ℃ for 14 days** | | **freeze-thaw cycles** | | **-80 ℃ for 30 days** | |
| --- | --- | --- | --- | --- | --- | --- | --- | --- | --- |
|  |  | **LQC** | **HQC** | **LQC** | **HQC** | **LQC** | **HQC** | **LQC** | **HQC** |
| Meropenem | Accuracy(%) | 110.42 | 110.76 | 113.02 | 108.42 | 108.37 | 108.97 | 113.82 | 102.56 |
|  | RSD(%) | 2.30 | 3.10 | 1.37 | 3.20 | 2.57 | 3.10 | 1.16 | 4.44 |
| Norvancomycin | Accuracy(%) | 104.78 | 105.13 | 107.44 | 108.62 | 102.27 | 110.14 | 108.29 | 106.28 |
|  | RSD(%) | 5.60 | 4.37 | 2.56 | 3.86 | 0.62 | 1.49 | 5.24 | 8.99 |
| Ampicillin | Accuracy(%) | 105.64 | 109.41 | 104.73 | 106.16 | 94.47 | 108.60 | 103.91 | 103.79 |
|  | RSD(%) | 1.52 | 1.41 | 1.59 | 1.62 | 1.54 | 5.33 | 1.54 | 5.35 |
| Linezolid | Accuracy(%) | 111.24 | 106.87 | 113.02 | 105.96 | 105.69 | 105.15 | 106.49 | 96.13 |
|  | RSD(%) | 3.33 | 1.30 | 0.79 | 2.79 | 1.58 | 1.16 | 12.88 | 3.63 |
| Ceftazidime | Accuracy(%) | 90.36 | 103.00 | 91.97 | 108.53 | 93.64 | 111.70 | 90.94 | 106.35 |
|  | RSD(%) | 4.66 | 3.13 | 1.32 | 2.82 | 1.85 | 1.62 | 3.69 | 1.61 |
| Cefriaxone | Accuracy(%) | 108.44 | 91.41 | 110.00 | 92.20 | 107.33 | 91.29 | 110.00 | 92.10 |
|  | RSD(%) | 3.09 | 2.29 | 2.64 | 2.00 | 3.88 | 6.29 | 1.21 | 4.27 |
| Daptomycin | Accuracy(%) | 98.80 | 107.22 | 106.16 | 110.10 | 99.71 | 110.22 | 105.53 | 108.26 |
|  | RSD(%) | 1.76 | 1.07 | 2.38 | 2.86 | 1.84 | 4.15 | 1.16 | 1.10 |
| Vancomycin | Accuracy(%) | 106.07 | 89.41 | 103.33 | 93.31 | 100.57 | 107.26 | 98.80 | 89.78 |
|  | RSD(%) | 2.32 | 6.57 | 13.93 | 2.40 | 6.96 | 0.72 | 9.32 | 1.01 |
| Cefepime | Accuracy(%) | 105.20 | 103.12 | 101.77 | 103.45 | 102.43 | 102.47 | 99.37 | 104.06 |
|  | RSD(%) | 3.65 | 1.13 | 5.90 | 2.83 | 2.69 | 2.90 | 11.50 | 5.22 |
| Biapenem | Accuracy(%) | 92.60 | 109.00 | 106.00 | 106.49 | 107.87 | 98.78 | 104.60 | 108.64 |
|  | RSD(%) | 2.92 | 3.85 | 3.67 | 4.72 | 2.20 | 3.46 | 1.16 | 3.72 |
| Cefoperazone | Accuracy(%) | 96.25 | 107.64 | 101.75 | 106.59 | 97.75 | 99.53 | 104.33 | 103.14 |
|  | RSD(%) | 5.74 | 0.29 | 4.90 | 4.34 | 1.93 | 2.04 | 3.04 | 2.44 |
| Latamoxef | Accuracy(%) | 96.33 | 109.57 | 108.81 | 111.09 | 107.44 | 109.93 | 109.92 | 104.83 |
|  | RSD(%) | 3.68 | 2.00 | 2.88 | 4.03 | 3.25 | 3.90 | 2.84 | 2.00 |
| Etimicin | Accuracy(%) | 112.50 | 107.82 | 86.40 | 91.42 | 107.23 | 110.58 | 87.10 | 90.50 |
|  | RSD(%) | 1.16 | 1.44 | 1.35 | 2.14 | 6.45 | 3.31 | 2.70 | 2.04 |
| Amikacin | Accuracy(%) | 108.20 | 104.16 | 93.14 | 96.72 | 105.70 | 103.36 | 93.16 | 99.00 |
|  | RSD(%) | 4.97 | 4.78 | 3.69 | 3.85 | 11.25 | 11.94 | 5.29 | 4.11 |

LQC: Low quality control; HQC: High quality control.

**Table S3** Stability of 14 antibiotics in human cerebrospinal fluid (n=3).

| **Compounds** | | **room temperature for 24 h** | | | **-20 ℃ for 8 days** | | **freeze-thaw cycles** | | **-80 ℃ for 30 days** | |
| --- | --- | --- | --- | --- | --- | --- | --- | --- | --- | --- |
|  |  | **LQC** | **HQC** | | **LQC** | **HQC** | **LQC** | **HQC** | **LQC** | **HQC** |
| Meropenem | Accuracy(%) | 107.33 | 107.54 | 107.36 | | 109.30 | 98.87 | 104.82 | 106.18 | 109.60 |
|  | RSD(%) | 2.43 | 4.64 | 0.20 | | 3.58 | 2.80 | 2.35 | 0.98 | 1.94 |
| Norvancomycin | Accuracy(%) | 102.33 | 107.85 | 93.47 | | 113.47 | 109.47 | 109.45 | 95.47 | 109.62 |
|  | RSD(%) | 1.67 | 2.06 | 2.08 | | 1.20 | 4.65 | 1.73 | 0.30 | 2.11 |
| Ampicillin | Accuracy(%) | 102.09 | 110.43 | 88.20 | | 108.39 | 95.07 | 112.52 | 87.31 | 109.45 |
|  | RSD(%) | 0.84 | 2.04 | 1.47 | | 1.31 | 2.04 | 7.35 | 1.31 | 1.47 |
| Linezolid | Accuracy(%) | 109.78 | 106.32 | 103.56 | | 104.17 | 100.67 | 101.91 | 110.27 | 108.20 |
|  | RSD(%) | 1.03 | 1.90 | 2.32 | | 1.76 | 1.43 | 0.84 | 1.83 | 2.72 |
| Ceftazidime | Accuracy(%) | 98.89 | 107.82 | 93.28 | | 113.05 | 96.86 | 108.30 | 93.33 | 112.37 |
|  | RSD(%) | 2.90 | 2.79 | 1.47 | | 1.30 | 0.50 | 2.68 | 2.17 | 1.42 |
| Cefriaxone | Accuracy(%) | 99.11 | 98.19 | 96.44 | | 88.67 | 102.89 | 89.17 | 100.22 | 99.89 |
|  | RSD(%) | 2.36 | 2.48 | 5.88 | | 3.93 | 4.69 | 2.88 | 2.52 | 9.01 |
| Daptomycin | Accuracy(%) | 107.02 | 110.52 | 98.04 | | 110.99 | 111.13 | 109.19 | 100.60 | 113.04 |
|  | RSD(%) | 0.59 | 3.34 | 0.70 | | 0.91 | 1.20 | 2.25 | 0.66 | 0.88 |
| Vancomycin | Accuracy(%) | 90.63 | 92.99 | 87.70 | | 94.25 | 110.77 | 90.56 | 107.67 | 98.56 |
|  | RSD(%) | 5.15 | 5.29 | 2.02 | | 0.99 | 4.86 | 3.72 | 0.43 | 1.89 |
| Cefepime | Accuracy(%) | 109.40 | 112.04 | 111.90 | | 110.63 | 113.63 | 108.63 | 101.27 | 112.86 |
|  | RSD(%) | 2.79 | 1.91 | 1.25 | | 2.92 | 0.18 | 4.12 | 0.91 | 0.56 |
| Biapenem | Accuracy(%) | 100.60 | 91.35 | 98.20 | | 96.98 | 95.60 | 95.91 | 108.87 | 106.91 |
|  | RSD(%) | 5.45 | 3.18 | 6.81 | | 1.89 | 3.12 | 2.38 | 4.13 | 2.66 |
| Cefoperazone | Accuracy(%) | 104.08 | 106.15 | 102.42 | | 107.17 | 93.08 | 111.66 | 107.17 | 100.58 |
|  | RSD(%) | 1.23 | 0.68 | 2.31 | | 1.04 | 3.91 | 1.02 | 0.57 | 0.60 |
| Latamoxef | Accuracy(%) | 108.14 | 111.38 | 104.97 | | 105.63 | 100.97 | 102.59 | 109.22 | 111.70 |
|  | RSD(%) | 3.18 | 1.80 | 3.71 | | 1.19 | 0.88 | 1.30 | 3.61 | 1.43 |
| Etimicin | Accuracy(%) | 99.04 | 94.41 | 96.71 | | 96.25 | 98.17 | 100.62 | 99.33 | 100.17 |
|  | RSD(%) | 1.42 | 1.71 | 3.53 | | 2.02 | 2.40 | 0.96 | 0.86 | 0.59 |
| Amikacin | Accuracy(%) | 90.23 | 105.56 | 91.02 | | 107.45 | 91.66 | 111.22 | 102.74 | 112.21 |
|  | RSD(%) | 0.20 | 0.90 | 0.13 | | 5.29 | 0.65 | 4.07 | 0.30 | 0.84 |

LQC: Low quality control; HQC: High quality control.


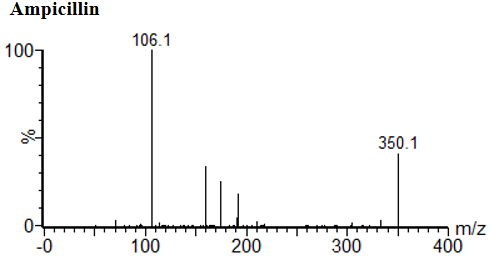

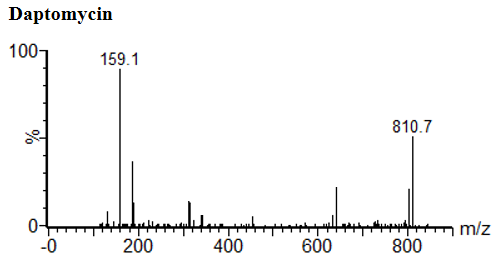

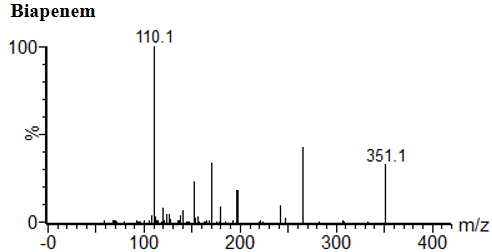

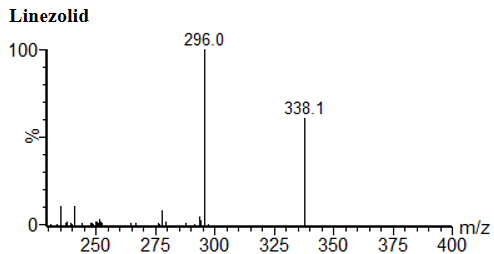

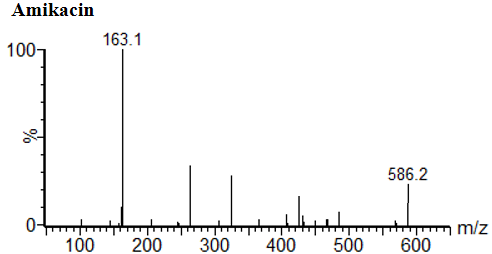

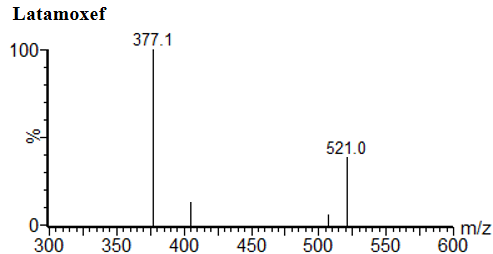

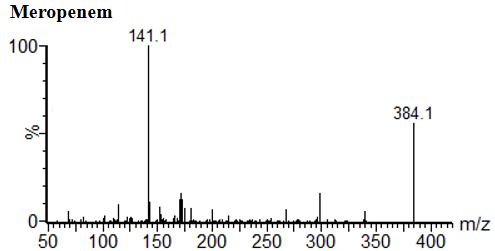

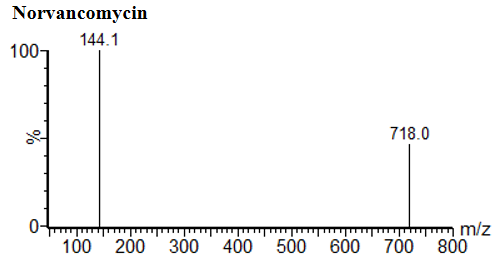

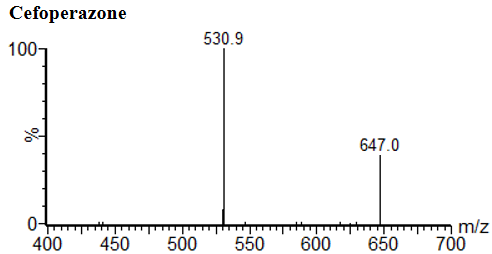

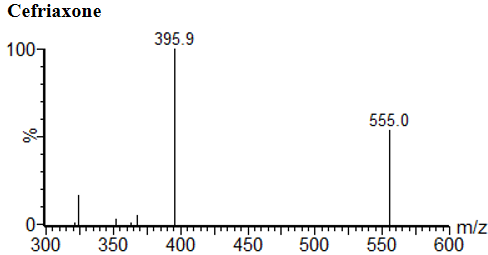

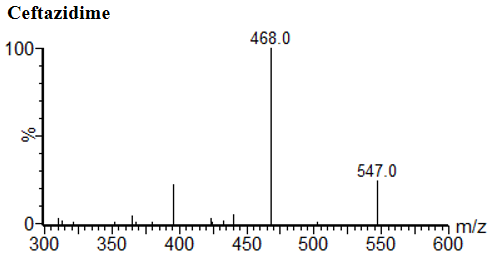

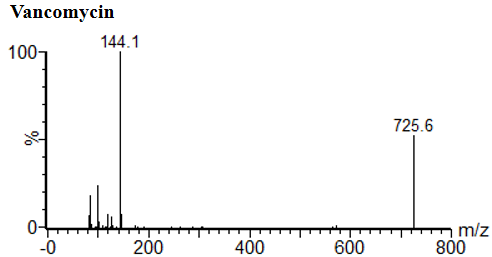

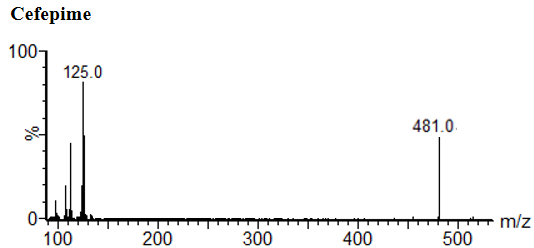

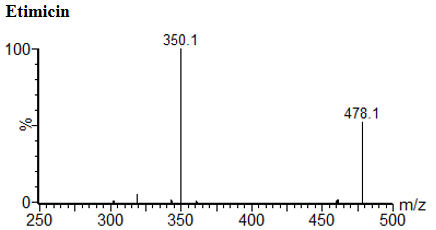


**Figure S1.** Acquisition parameters used in the UPLC-MS/MS assay.


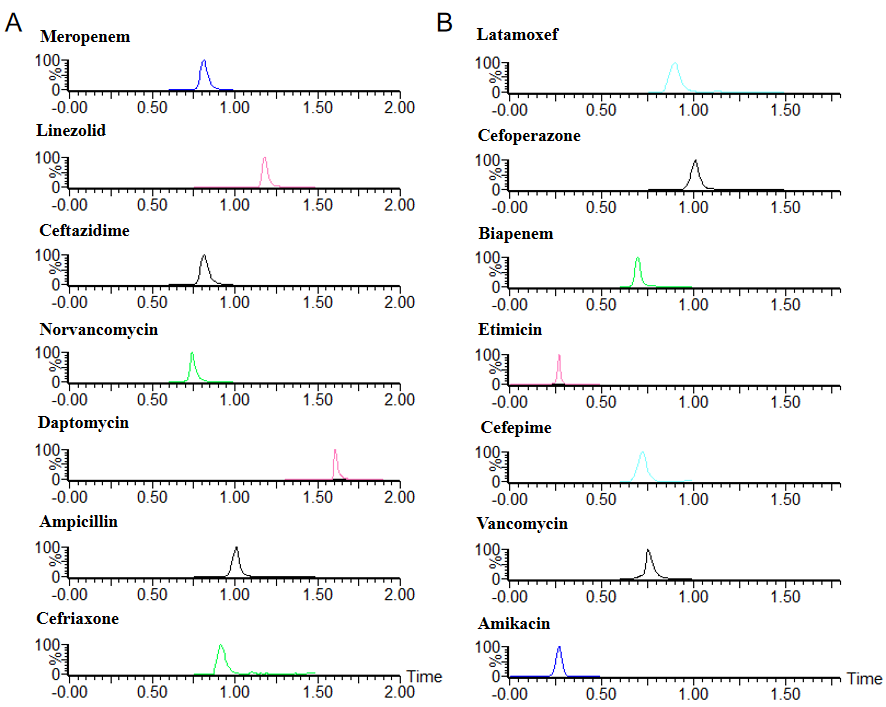


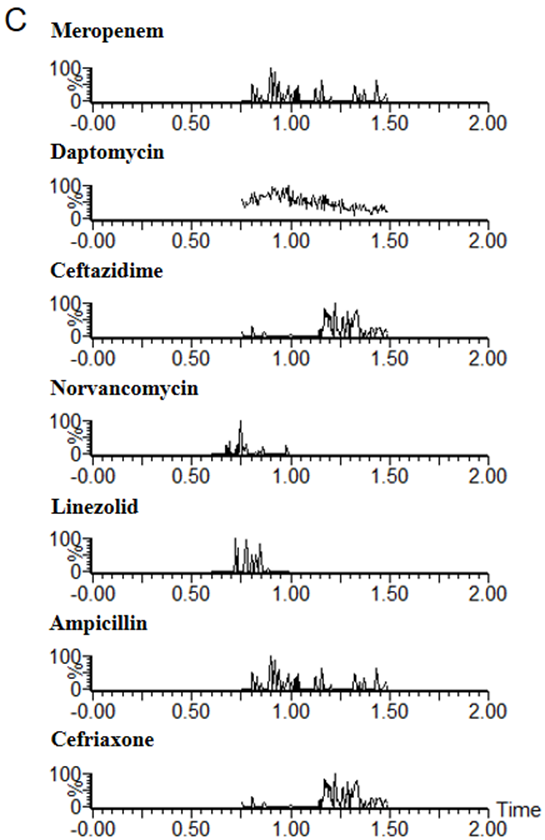

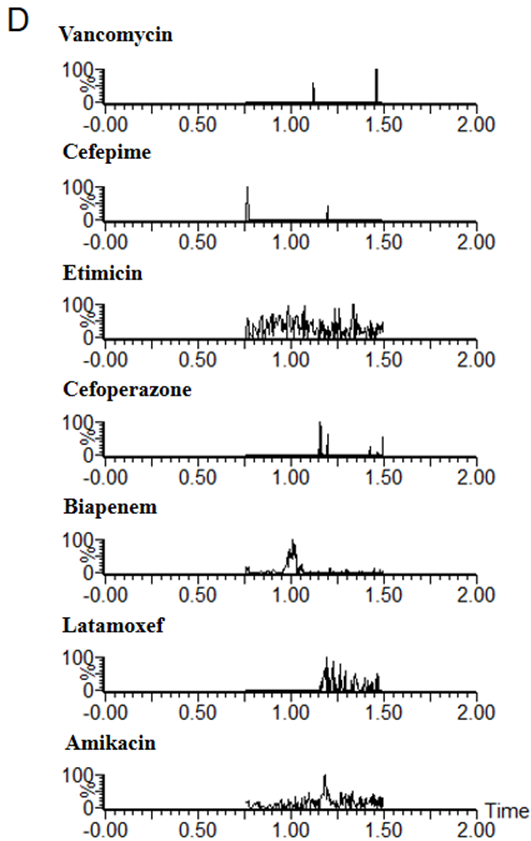

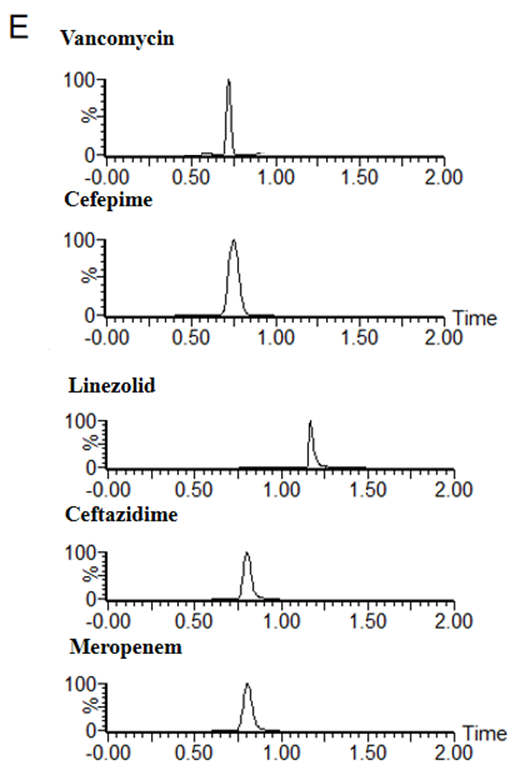


**Figure S2.** Representative multiple reaction monitoring chromatograms of analytes in cerebrospinal fluid. A and B, LLOQ; C and D, blank sample; E, patients samples.
